# Benefits of switching from intravenous to subcutaneous daratumumab: Perspectives from UK healthcare providers

**DOI:** 10.3389/fonc.2023.1063144

**Published:** 2023-02-23

**Authors:** Gordon Cook, John Ashcroft, Mariana Fernandez, Sarah Henshaw, Zeyad Khalaf, Guy Pratt, Anish Tailor, Neil Rabin

**Affiliations:** ^1^ Leeds Cancer Research UK Clinical Trials Unit, Leeds Institute of Clinical Trials Research, University of Leeds, Leeds, United Kingdom; ^2^ Department of Haematology, St James’s University Hospital, Leeds, United Kingdom; ^3^ Department of Haematology, Pinderfields General Hospital, Wakefield, United Kingdom; ^4^ Janssen Europe, the Middle East and Africa (EMEA), Beerse, Belgium; ^5^ Department of Haematology, Nottingham University Hospitals, Nottingham, United Kingdom; ^6^ Department of Haematology, Queen Elizabeth Hospital Birmingham, Birmingham, United Kingdom; ^7^ Department of Haematology, University College London Hospitals, London, United Kingdom

**Keywords:** daratumumab, intravenous, multiple myeloma, subcutaneous, quality of life

## Abstract

Daratumumab is a CD38-directed monoclonal antibody indicated to treat multiple myeloma (MM). Daratumumab was initially administered intravenously (IV), subsequently a subcutaneous (SC) formulation was developed to increase convenience of administration. The UK was an early adopter of SC daratumumab and, as such, this report provides consensus recommendations from a group of UK MM experts, with the aim of facilitating the transition from IV to SC daratumumab for other European healthcare providers. The switch from IV to SC daratumumab has been beneficial to patients and healthcare providers, as it simplifies treatment, reduces pressure on hospitals and can improve patients’ quality of life.

## Introduction

Multiple myeloma (MM) is a hematological malignancy of plasma cells initiating in bone marrow (1). Despite an increase in the number of treatment options for patients with MM, it remains incurable with a relapsing remitting course until the relapsed/refractory (R/R) stage, where outcomes remain poor (1).

Daratumumab (DARA) is a first-in-class CD38-directed monoclonal antibody indicated as a monotherapy and in combination regimens for the treatment of patients with MM (2–5). DARA was initially administered as an intravenous (IV) infusion, which takes approximately 7 hours at first infusion and 3–4 hours at each subsequent infusion (4–6). A rapid IV regimen in which DARA is infused over a 90-minute period has also been investigated, but remains unlicensed (4, 5, 7, 8). IV DARA is associated with infusion-related reactions (IRRs) in 37% of patients at first infusion (5).

To increase the convenience of DARA administration, an 1800 mg flat-dose SC formulation was developed, which is administered over 3–5 minutes (2, 3, 9). It has been demonstrated that SC DARA is well tolerated in combination with standard regimens and as a monotherapy, with low rates of IRRs (9–11). The Phase II PLEIADES study showed that SC DARA in standard-of-care regimens has similar clinical efficacy to IV DARA (11). The Phase III COLUMBA study demonstrated the non-inferiority of SC DARA compared with IV DARA (overall response seen in 108 [41%] patients in the SC group and 96 [37%] in the IV group), and these benefits were seen alongside reduced administration times (9). A recent analysis of the COLUMBA study suggested greater treatment satisfaction in patients receiving SC DARA versus IV DARA (12). SC DARA is also associated with less active healthcare provider (HCP) time compared with IV DARA (total active HCP time reduced by 63.8% and 49.5% with DARA SC for first and subsequent doses, respectively) (13).

The UK was an early adopter of SC DARA, and as such a group of UK-based MM experts were convened to provide consensus recommendations for switching from IV to SC DARA, including patient perspectives. The aim of this report is to facilitate the transition from IV to SC DARA for other European HCPs.

## Drivers for switching from IV to SC DARA

Experts recognise that the drivers for switching from IV to SC DARA include patient convenience and the potential for improving efficiencies in healthcare settings. A recent systematic review of SC oncology biologics found convenience of administration and health-related quality of life (HRQoL) are improved with SC over IV formulations (14).

Patient-reported satisfaction was recently analyzed in 529 patients with R/R MM who received DARA IV or DARA SC in the Phase III COLUMBA study (12). In this study, SC DARA was associated with greater patient satisfaction with treatment compared with IV DARA. Furthermore, treatment satisfaction with DARA SC was maintained over the long term (through cycle 10), a noteworthy point for patients with R/R MM who require long-term treatment to relieve disease symptoms and establish disease control (12). The authors suggest that patient satisfaction with DARA SC may be related to shorter administration times and lower rates of IRRs.

SC DARA was also associated with less active HCP time during preparation and administration than IV DARA (13).

In addition, the SC formulation would move DARA administration outside of hospitals, helping to reduce hospital footfall during the COVID-19 pandemic and provide long-term optimization of day unit capacity. The authors also note that SC DARA was associated with less active HCP time during preparation and administration than IV DARA.

## Preparation of SC DARA

The expert consensus was that SC DARA can be easily and safely prepared on the ward or in the patient’s home, with the maintenance of aseptic technique, reducing the burden on the pharmacy compared with IV DARA. Crucially, the experts agree that the preparation of SC DARA requires appropriate standard operating procedures, including a monoclonal antibody risk assessment and HCP education.

Experts agreed that the procedure for preparing SC DARA is more streamlined compared with IV DARA, as pre-medications can be taken orally by patients prior to visiting the hospital. Furthermore, the similarities between preparing SC DARA and other SC drugs used in hematology may mean that nurses already have experience with similar preparations.

In addition, all patients receive the same dose (1800 mg) when DARA is administered by SC injection, whereas IV doses vary according to the patient’s weight (2–5). This uniform dosing facilitates stocking of DARA within clinics/pharmacies.

Finally, SC injections are easier to deliver than IV infusions: skilled personnel are not required, the injections are less painful, and the risk of infection is lower (15).

## Administration and monitoring of SC DARA

In contrast to IV DARA, SC DARA is administered at a flat-dose of 1800 mg (9). Median weight in the SC DARA arm of the Phase III COLUMBA trial (NCT03277105) was 72.4 kg (range: 39.0–130.0) (9). There are limited data on SC DARA in patients with a body weight of >120 kg and, hence, no dose modifications are recommended for this patient population (3). The experts also raised the possibility of potential over-treatment of patients with low body weight, although they believed that in clinical practice this would only apply to a small number of individuals. Higher rates of neutropenia were observed in patients with a body weight of ≤65 kg, but as this did not lead to higher rates of serious infection, no dose modifications are recommended (3, 9). The experts noted there may be practical difficulties of administering SC DARA in patients with low body weight, with difficulty pinching the skin and maintaining that pinch in order to give the injection over 3–5 minutes. The experts are currently unaware of any other comorbidity-related issues in switching from IV to SC DARA.

**Figure 1:**
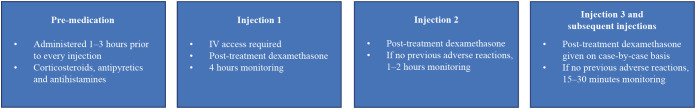
Recommendations for pre-medication, administration and post-administration monitoring of SC DARA. DARA, daratumumab; IV, intravenous; SC, subcutaneous.

At 1–3 hours prior to administration of each SC DARA injection, patients should be given corticosteroids, antipyretics and antihistamines (3). IV access is required (as per local recommendations) with the first injection of SC DARA in case of a serious infusion reaction. There was consensus that 4 hours of post-injection monitoring is necessary after the first injection of SC DARA (**Figure 1**). Patients do not need to stay in their chair continuously during this period but should be monitored every 15 minutes during the first 30 minutes, at 1 hour and then hourly. Real-world data suggest that the observation time can be reduced for doses 2 and 3, and no observation is needed after the fourth dose (16). This recommendation is relevant both for patients new to DARA and those who are switching from IV to SC.

In general, the experts agreed that dexamethasone should be given after the first, and possibly second, injection of SC DARA to mitigate the risk of potential reactions (**Figure 1**). Each patient should be reviewed individually to determine if they should receive corticosteroids with subsequent injections (**Figure 1**). Retrospective studies have shown that patients treated with montelukast prior to the first DARA IV infusion experience a reduction in IRRs (17, 18), additional studies to determine the benefits of montelukast treatment prior to DARA SC are required.

Patients should be educated about the possibility of local reactions following SC DARA. The injection site for SC DARA (the abdomen approximately 7.5 cm to the right or left of the navel) should be rotated for successive treatments (3). Adverse reactions after the third injection of SC DARA had not been seen by the experts, but collection of further data on the incidence and timing of injection reactions is important to inform future practice.

## Patient perspectives on SC DARA

The experts believe that a majority of patients are willing to switch from IV to SC DARA. Patients understand the benefits that switching will bring them, e.g. less time spent in hospital (estimated treatment time for SC DARA was reduced by 97% versus IV DARA at first and subsequent treatments) (13), and increased patient satisfaction with SC compared with IV DARA reported in the COLUMBA trial (12). Patient satisfaction is especially important given the continuous nature and long duration of treatment (12).

The minority of patients who might not want to switch are those who have achieved disease control on IV DARA (and may not have done so with other treatments), needle-phobic patients and rare patients who have skin conditions or significant oedema.

## Flexible care in hematological oncology

The concept of flexible care in hematological oncology (i.e. delivering treatment at home or outside of the hospital setting) became increasingly important during the COVID-19 pandemic, as the trend towards remote care has accelerated (19). The delivery of SC drugs, including DARA, in the community reduces the travel burden for patients and their caregivers compared with IV treatments (19).

The next challenge in the UK will be to translate SC DARA into the community setting. There are geographical limitations to outreach delivery and a combination approach may be needed to cover urban and rural areas. There are also financial and resource challenges, as delivering therapies at home is time-intensive for nurses. The ‘mobile chemotherapy unit’ model of service delivery can be used for other services and may be more financially viable than delivering SC DARA alone. Proper logistical planning of outreach services is challenging but essential to maximize efficiency, for example, through sequential treatment of patients in the same area.

The experts anticipate that digital data collection, for example biometric data collected from wearable devices, accompanied with patients’ own experiences, are likely to play an increasing role in the future of flexible care. However, the use of these technologies needs to be carefully assessed in the context of both safety and providing a true utility and benefit above and beyond our present standards of care.

## Self-administration of SC DARA

Self-administration of monoclonal antibodies is well established in disease areas such as rheumatoid arthritis and multiple sclerosis (20), but is not yet common practice in oncology due to safety concerns and the need to administer relatively large dosing volumes (20). Although self-administration of DARA is not currently on label, it may be an option for some patients in the future.

It is important to assess any self-administrating device in a real-world setting, and to include a diverse group of patients, not just the highly motivated patients attracted to participate in clinical trials. The feasibility of such a device, potential benefits to the healthcare system and HRQoL data should be assessed. There was consensus between the experts that not all patients would be able or willing to self-administer SC DARA. Patients would need to inject 15 ml of liquid over 3–5 minutes (3), and thus would need suitable dexterity, confidence and education. **Table 1** summarizes the recommendations from the experts on the self-administration of SC DARA.

**Table 1:** Recommendations on the self-administration of SC DARA.

A reliable and user-friendly administration device would be required to facilitate the successful self-administration of SC DARA. This would ideally be pre-loaded, so that the patient does not need to prepare anything.
Some hospital trusts do not, at present, have the capacity to enable changes in treatment delivery and should be supported to allow innovative ways of delivering chemotherapy in patients’ homes. This would include patient education support, safe delivery and disposal of medication, and appropriate reimbursement.
In urban areas, treatment delivery is likely to be carried out by HCPs. Self-administration may be more important for rural communities.
Some patients may not want to administer an injection, therefore self-administration could increase the stress of treatment. Many patients prefer an HCP to administer their treatments. Patients should be supported if this is their choice.

DARA, daratumumab; HCP, healthcare provider; SC, subcutaneous.

## Conclusion

Switching from IV to SC DARA is simpler for both patients and HCPs, reduces pressure on hospitals and aseptic units, and can increase HRQoL. The development of standardized protocols and risk assessments for the preparation and administration of SC DARA, in addition to guidelines for post-treatment monitoring, will help treatment centers transition from IV to SC DARA. Flexible care and home delivery in hematological oncology, which has been accelerated by the COVID-19 pandemic, is seen as the future of cancer treatment, and represents an opportunity to further decrease the burden of treatment on patients’ lives.

## Data availability statement

The original contributions presented in the study are included in the article/supplementary material. Further inquiries can be directed to the corresponding author.

## Author contributions

On 6 April 2021, six healthcare providers (GC, JA, SH, GP, AT and NR) from the UK and two Janssen employees (MF and ZK) met to discuss their experiences of switching patients from IV to SC daratumumab, which is the basis for this consensus document. ZK was an employee of Janssen at the time of the meeting, and during the majority of manuscript development, and has since moved to GSK. All authors confirm that they meet the International Committee of Medical Journal Editors (ICJME) requirements for authorship and that they have contributed to drafting/critically revising the article and sharing in the final responsibility for the content of the manuscript and the decision to submit it for publication. All authors contributed to the article and approved the submitted version.
